# How Could Future AI Help Tackle Global Complex Problems?

**DOI:** 10.3389/frobt.2020.00050

**Published:** 2020-04-21

**Authors:** Anne-Marie Grisogono

**Affiliations:** College of Science and Engineering, Flinders University, Adelaide, SA, Australia

**Keywords:** AI decision support, complex decisions, human limitations, wicked problems, interface design

## Abstract

How does AI need to evolve in order to better support more effective decision-making in managing the many complex problems we face at every scale, from global climate change, collapsing ecosystems, international conflicts and extremism, through to all the dimensions of public policy, economics, and governance that affect human well-being? Research in complex decision-making at an individual human level (understanding of what constitutes more, and less, effective decision-making behaviors, and in particular the many pathways to failures in dealing with complex problems), informs a discussion about the potential for AI to aid in mitigating those failures and enabling a more robust and adaptive (and therefore more effective) decision-making framework, calling for AI to move well-beyond the current envelope of competencies.

## Introduction

Human intelligence rests on billions of years of evolution from the earliest origins of life, and despite its undeniably unique nature within the biosphere, and the apparent gulf that distinguishes the human species from all others, it should nevertheless be seen as an extremum within a continuum. The unifying feature of all natural intelligence systems is that they have evolved under strong selection pressures to solve the problems of surviving and thriving sufficiently to reproduce better than their competitors. Unlike the evolution of faster speed, sharper teeth, more efficient energy harvesting and utilization, or better camouflage, all of which improve physical capabilities, the evolution of intelligence enables better choices to be made as to how and when to employ those capabilities, by processing relevant sensed and stored information. If the environment is challenging enough, whether through the prevalence of threats, the scarcity of necessary resources or through intense competition for them, then there is a high fitness pay-off for evolving both the necessary physical characteristics for sensing, processing and storing the relevant information, and the intelligence to exploit them.

From this perspective we can define intelligence as the ability to produce effective responses or courses of action that are solutions to complex problems—in other words, problems that are unlikely to be solved by random trial and error, and that therefore require the abilities to make finer and finer distinctions between more and more combinations of relevant factors and to process them so as to generate a good enough solution. Obviously this becomes more difficult as the number of possible choices increases, and as the number of relevant factors and the consequence pathways multiply. Thus complexity in the ecosystem environment generates selection pressure for effective adaptive responses to the complexity.

One possible adaptive strategy is to find niches to specialize for, within which the complexity is reduced. The opposite strategy is to improve the ability to cope with the complexity by evolving increased intelligence at an individual level, or collective intelligence through various types of cooperative or mutualistic relationships. Either way, increased intelligence in one species will generally increase the complexity of the problems they pose for both other species in the shared ecosystem environment, and for their own conspecifics, driving yet further rounds of adaptations. Even when cooperative interactions evolve to deal with problems that are more complex than an individual can cope with, the shared benefits come with a further complexity cost in maintaining the cooperative relationships and policing for cheats (Nowak, 2006).

This ratcheting dynamic of increasing intelligence and increasing complexity continues as long as two conditions are met: further increases in sensing and processing are sufficiently accessible to the evolutionary process, and the selection pressure is sufficient to drive it. Either condition can fail. Thus generally a plateau of dynamic equilibrium is reached. But it is also possible that under the right conditions, which we will return to below, the ratcheting of both complexity and intelligence may continue and accelerate.

Artificial intelligence on the other hand, has not evolved through natural selection, but rather owes its genesis to human intelligence (at least on this planet), which has a number of important implications that have colored its trajectory so far. But to contemplate its possible futures and ours, this paper argues the need to re-examine the relationship between human and machine within a much broader context. In particular, we need to understand both the strengths and the limitations of human intelligence, consider what our most pressing issues are and what kinds of advances in AI would be most useful in helping us to navigate those complex problems in the near to mid-term. At the same time we need to be mindful of the risks, not only in the nearer term but also those that may only materialize as longer term consequences, and address how these may be averted or mitigated.

## AI and the Limitations of Human Intelligence

It was natural for the pioneers of AI to choose human cognitive abilities such as playing chess or Go, navigating obstacles, or recognizing and interpreting written and spoken language, as the yardsticks by which to measure early progress in AI capabilities, not only because they were so far beyond what could be simulated at the time, but also perhaps, because we felt so impressed with our own dazzling cognitive strengths. But now that many of these and other quintessentially human examples of intelligence are being relegated to the growing list of tasks at which AI can surpass human performance, we need to step back and acknowledge that human intelligence is not the pinnacle of what can be achieved.

Just as the Copernican revolution and later astronomical discoveries dislodged us from the center of the universe and pushed us into orbiting a minor star in an undistinguished galaxy, and Darwinism pushed us from the pre-eminent position we had assumed over all life forms into just a twig of the evolutionary tree of life, the current and recent sweep of advances in understanding of neuroscience, cognition, behavioral science, evolutionary psychology and related fields call for yet another round of humbling re-appraisal of where we fit in the grand scheme of things.

Taking the concept of intelligence as the ability to produce effective solutions to complex problems by processing relevant sensed and stored information, it is evident that human intelligence and ingenuity have led to immense progress in producing solutions for many of the pressing problems of past generations, such as higher living standards, longer life expectancy, better education and working conditions. But it is equally evident that the transformations they have wrought in human society and in the planetary environment include many harmful unintended consequences, and that the benefits themselves are not equitably distributed and have often masked unexpected downsides.

We are now confronting a complex network of interdependent global problems which we seem increasingly incapable of dealing with effectively at either the national or international levels, and arguably it is the very successes of human intelligence that have ratcheted the complexity of the challenges we face to a level that unaided human intelligence is now unable to cope with.

This was recognized as long ago as 1973 in a remarkably prescient paper (Rittel and Webber, 1973) in which the authors coined the term “wicked problems” (as opposed to benign problems which are tractable) and laid out ten hallmarks^1^ characterizing them, together with a very clear analysis of their roots in complexity. Their inability to lay out an equally clear prescription for the resolution of such wicked problems signaled that a tipping point had indeed been reached where our limitations had now outstripped our cleverness.

What has changed in the intervening decades? While the scale and urgency of the global problems we face have certainly intensified, what we have since learned in the germane fields of complexity science, evolutionary psychology, brain and behavioral science, and artificial intelligence, suggests that we may be close to another tipping point where we could possibly drive the emergence of advanced artificial intelligence systems that can effectively support human decision-making in managing such problems, by a combination of mitigating human fallibilities and complementing human shortcomings.

At this point the reader may be wondering why there should be a human in the decision process at all if we have indeed overstepped our domain of competence. There are possibly three reasons.

Firstly, even if there does come a day when AI systems are judged able to take over the management of complex issues without human control, such a judgment would imply that humans have confidence in those systems, and such confidence can only be developed through a transition period of human and machine working together, learning the strengths and limits of each other's capabilities, and evolving better ways to arrive at good decisions, through evaluating and learning from the consequences of those decisions. Secondly, there is the perennial issue of expert knowledge elicitation. Despite all their human failings, there is surely a vast, unquantifiable reservoir of relevant experiential implicit knowledge, and hopefully wisdom, in the cohorts of public officials, managers and analysts who currently strive to deal with these spiraling problems. If an AI system is to eventually run things without them, it had better somehow absorb what they know that cannot be itemized in databases - which links to the third reason: will people really want to be excluded from managing their societies and enterprises? The answer that might emerge in the future when the question actually becomes pertinent is impossible to predict today. But we have enough reasons to proceed on the assumption that the next steps will involve advanced AI support for human decision-makers.

To propose a set of desiderata for the advances in AI that are needed we now turn to what we have learned about the specific limitations that plague human decision-makers in complex problems. We can break this down into two parts: the aspects of complex problems that we find so difficult, and what it is about our brains that limits our ability to cope with those aspects.

### Sources of Difficulty in Complexity

***Interdependence*** is a defining feature of complexity and has many challenging and interesting consequences. In particular, the network of interdependencies between different elements of the problem means that it cannot be successfully treated by dividing it into sub-problems that can be handled separately. Any attempt to do that creates more problems than it solves because of the interactions between the partial solutions.

Dynamical processes driving development of the situation often involve many positive and negative feedbacks, thus amplifying and suppressing different aspects of the situation, and resulting in highly ***non-linear dynamics***. This means that relying on linear extrapolation of current conditions can lead to serious errors.

There is no natural boundary that completely isolates a complex problem from the context it is embedded in. There is always some traffic of information, resources, and agents in and out of the situation which can bring about unexpected changes, and therefore the ***context*** ***cannot be excluded*** from attention.

Complex problems exist at ***multiple scales***, with different agents, behaviors and properties at each, but with interactions between scales. This includes both emergence, the appearance of complex structure and dynamics at larger scales as a result of smaller-scale phenomena, and its converse, top-down causation, whereby events or properties at a larger scale can alter what is happening at the smaller scales. In general, all the scales are important, there is no single “right” scale at which to act.

Interdependence implies multiple interacting causal and influence pathways leading to, and fanning out from, any event or property, so simple causality (one cause—one effect), or linear causal chains will not hold in general. Yet much of our cultural conditioning is predicated on a naïve view of linear causal chains, such as finding “the cause” of an effect, or “the person” to be held responsible for something, or “the cure” for a problem. Focusing on singular or primary causes makes it more difficult to intervene effectively in complex systems and produce desired outcomes without attendant undesired ones—so-called “side-effects” or ***unintended*** ***consequences***. Effective decision making requires the ability to develop sufficient understanding of the causal and influence network to engage with it effectively, neither oversimplifying it, nor becoming overwhelmed with unnecessary levels of detail.

Furthermore, such networks of interactions between contributing factors can produce ***emergent behaviors*** which are not readily attributable or intuitively anticipatable or comprehensible, implying unknown risks and unrecognized opportunities.

There are generally ***multiple interdependent goals*** in a complex problem, both positive and negative, poorly framed, often unrealistic or conflicted, vague or not explicitly stated, and stakeholders will often disagree on the weights to place on the different goals, or change their minds. Achieving sufficient high level goal clarity to develop concrete goals for action is in itself a complex problem.

Complex situations generally contain many ***adaptive agents*** with complex relationships and shifting allegiances, and new behaviors and features continually arise. This means that approaches that worked in the past may no longer work, interventions that frustrate the intents of some agents will often simply stimulate them to find new ways to achieve them, and opportunities created by the inevitable new vulnerabilities that interventions create will be rapidly identified and exploited.

Many important aspects of complex problems are hidden, so there is ***inevitable*** ***uncertainty*** as to how the events and properties that are observable, are linked through causal and influence pathways, and therefore many hypotheses about them are possible. These cannot be easily distinguished based on the available evidence.

### Limitations of the Human Brain

The brief overview above reveals some of the cognitive abilities that are essential for successful tackling of complex problems. One immediate conclusion that can be drawn is that there is a massive requirement for cognitive bandwidth—not only to keep all the relevant aspects at all the relevant scales in mind as one seeks to understand the nature of the problem and what may be possible to do, but even more challenging, to incorporate appropriate non-linear dynamics as trajectories in time are explored. Given the well-known limitations of human working memory, short-term memory and attention span, this is an obvious area for advanced AI support to target.

But there is a more fundamental problem that needs to be addressed first: how to acquire the necessary relevant information about the composition, structure and dynamics of the complex problem and its context at all the necessary scales, and revise and update it as it evolves. This requires a stance of continuous learning, i.e., simultaneous sensing, testing, learning and updating across all the dimensions and scales of the problem, and the ability to discover and access relevant sources of information. At their best, humans are okay at this, up to a point, but not at the sheer scale and tempo of what is required in real world complex problems which refuse to stand still while we catch up.

Moreover, there are both physiological factors such as the impacts of stress, fatigue and anxiety on cognitive performance, and particular features of the human brain, legacies of our evolutionary history, which compound the difficulties.

Because the human brain evolved to deal with the problems of surviving and thriving that our ancestors faced, modern humans are still equipped with the same heuristics, behavioral tendencies and biases that worked well enough in the distant past. These hardwired shortcuts based on rules of thumb, operating automatically below conscious awareness and so permitting very rapid adaptive responses to various simple conditions, enabled them to cope with the level of complexity that existed then—keeping track of a hundred or so individuals and their interactions, intents, and histories (Dunbar, 1992). But features relying on approximations that held true for dealing with common problems in past environments can morph into risky bugs in today's highly interconnected and rapidly evolving complex situations (Kahneman, 2002).

To understand how all these factors interact to limit human competence in managing complex problems, and what opportunities might exist for mitigating them through advanced AI systems, we now review some key findings from relevant research.

In particular we are interested in learning about the nature of human decision-making in the context of attempting to manage an ongoing situation which is sufficiently protracted^2^ and complex to defeat most, but not all^3^, decision-makers. Drawing useful conclusions about the detailed decision-making behaviors that tend to either sow the seeds of later catastrophes, or build a basis for sustained success, calls for an extensive body of empirical data from many diverse human subjects making complex decisions in controllable and repeatable complex situations. Clearly this is a tall ask, so not surprisingly, the field is sparse. However, one such research program (Dörner, 1995; Evans et al., 2011; Dörner and Gerdes, 2012; Dorner and Güss, 2013; Donovan et al., 2015), which has produced important insights about how successful and unsuccessful decision-making behaviors differ, stands out in having also addressed the underlying neurocognitive and affective processes that conspire to make it very difficult for human decision-makers to maintain the more successful behaviors, and to avoid falling into a vicious cycle of less effective behaviors.

In brief, through years of experimentation with human subjects attempting to achieve complex goals in computer-based micro-worlds with complex underlying dynamics, the specific decision-making behaviors^4^ that differentiated a small minority of subjects who achieved acceptable outcomes in the longer term, from the majority who failed to do so, were identified. Results indicated that most subjects could score some quick wins early in the game, but as the unintended consequences of their actions developed and confronted them, and their attempts to deal with them created further problems, the performance of the overwhelming majority (~90%) quickly deteriorated, pushing their micro-worlds into catastrophic or chronic failure.

As would be expected, their detailed behaviors reproduced many well-documented findings about the cognitive traps posed by human heuristics and biases. Low ambiguity tolerance was found to be a significant factor in precipitating the behavior of prematurely jumping to conclusions about the problem and what was to be done about it, when faced with situational uncertainty, ambiguity and pressure to achieve high-level goals. The chosen (usually ineffective) course of action was then defended and persevered with through a combination of confirmation bias (Nickerson, 1998), commitment bias (Staw, 1997), and loss aversion (Kahneman and Tversky, 1979), in spite of available contradictory evidence. The unfolding disaster was compounded by a number of other reasoning shortcomings such as difficulties in steering processes with long latencies and in projecting cumulative and non-linear processes (Sterman, 1989). Overall they had poor situation understanding, were likely to focus on symptoms rather than causal factors, were prone to a number of dysfunctional behavior patterns, and attributed their failures to external causes rather than learning from them and taking responsibility for the outcomes they produced.

By contrast, the remaining ten percent who eventually found ways to stabilize their micro-world, showed systematic differences in their decision-making behaviors and were able to counter the same innate tendencies by taking what amounts to an adaptive approach, developing a conceptual model of the situation, and a stratagem based on causal factors, seeking to learn from unexpected outcomes, and constantly challenging their own thinking and views. Most importantly, they displayed a higher degree of ambiguity tolerance than the unsuccessful majority.

These findings are particularly significant here because most of the *individual* human decision-making literature has concentrated on how complex decision-making fails, not on how it succeeds. However, insights from research into successful *organizational* decision-making in complex environments (Collins, 2001; Weick and Sutcliffe, 2001), do corroborate the importance of taking an adaptive approach.

In summary, analysis of the effective decision behaviors offers important insights into what is needed, in both human capabilities and AI support, to deal with even higher levels of complexity beyond current human competence. There are two complementary aspects here—put simply: how to avoid pitfalls (what not to do), and how to adopt more successful approaches (what to do instead).

It is not difficult to understand how the decision making behaviors associated with the majority contributed to their lack of success, nor how those of the rest enabled them to develop sufficient conceptual and practical understanding to manage and guide the situation to an acceptable regime. Indeed if the two lists of behaviors are presented to an audience, everyone can readily identify which list leads to successful outcomes and which leads to failure. Yet if those same individuals are placed in the micro-world hot seat, 90% of them will display the very behaviors they just identified as likely to be unsuccessful. This implies that the displayed behaviors are not the result of conscious rational choice, but are driven to some extent by unconscious processes.

This observation informed development of a theoretical model (Dörner and Gerdes, 2012; Dorner and Güss, 2013) incorporating both cognitive and neurophysiological processes to explain the observed data. In brief, the model postulates two basic psychological drives that are particularly relevant to complex decision making, a need for certainty and a need for competence. These are pictured metaphorically as tanks which can be topped up by signals of certainty (one's expectations being met) and signals of competence (one's actions producing desired outcomes), and drained by their opposites—surprises and unsuccessful actions. The difference between the current level and the set point of a tank creates a powerful unconscious need, stimulating some behavioral tendencies and suppressing others, and impacting on cognitive functions through stimulation of physiological stress. If both levels are sufficient the result is motivation to explore, reflect, seek information and take risky action if necessary—all necessary components of effective decision making behavior. But if the levels get too low the individual becomes anxious and is instead driven to flee, look for reassurance from others, seek only information that confirms his existing views so as to top up his dangerously low senses of certainty and competence, and deny or marginalize any tank-draining contradictory information. The impacts of stress on cognitive functions reinforce these tendencies when the levels are too low by reducing abilities to concentrate, sustain a course of action, and recall relevant knowledge.

Individuals whose tanks are low therefore find it difficult to sustain the decision-making behaviors associated with success, and are likely to act in ways that generate further draining signals, digging themselves deeper into a vicious cycle of failure. We can now understand the 90:10 ratio, as the competing attractors are not symmetric—the vicious cycle of the less effective decision behaviors is self-reinforcing and robust, while the virtuous cycle of success is more fragile because one's actions are not the sole determinant of outcomes in a complex situation, so even the best decision-makers will sometimes find their tanks getting depleted, and therefore have difficulty sustaining the more effective decision making behaviors.

Further research has demonstrated that the more effective decision making behaviors are trainable to some extent, but because they entail changing meta-cognitive habits they require considerable practice, reinforcement and ongoing support (Evans et al., 2011; Grisogono and Radenovic, 2011; Donovan et al., 2015). However, the scope for significant enhancement of unaided human complex decision making competence is limited—not only in the level of competence achievable, but also and more importantly, in the degree of complexity that can be managed.

Meanwhile, the requirements for increased competence, and the inexorable rise in degree of complexity to be managed, continue to grow.

### How Could AI Help?

Recent AI advances such as deep learning and generative adversarial networks have demonstrated impressive results in many domains—superhuman precision in classification tasks, beating human world champions in Go, and generation of images that are hard for humans to discriminate from reality, to name a few.

But what are the prospects for advances in AI to deliver the kind of decision support capability that is needed by those charged with managing the most challenging, indeed wicked, problems? And can those advances be achieved by research that continues to set goals based on beating human performance, or on fooling human discrimination?

Despite its successes, the best examples of AI are still very specialized applications that focus on well-defined domains, and that generally require a vast amount of training data to achieve their high performance. Such applications can certainly be components of an AI decision support system for managing very complex problems, but the factors discussed in the two previous sections imply that much more is needed: not just depth in narrow aspects, but breadth of scope by connecting the necessary components so as to create a virtual environment which is a sufficiently valid model of the problem and its context, and in which decision-makers can safely explore and test options for robustness and effectiveness, while being supported in maintaining effective decision making behaviors and resisting the less effective ones. The following section develops a more detailed set of desiderata for such an AI support system.

The resurgence of interest in Artificial General Intelligence seems a promising avenue for the kinds of advances that are needed, but it is telling that AGI is most often explicitly pursued through the lens of the touted general intelligence that humans possess (Adams et al., 2012), in other words still focusing on what we believe we are good at, rather than exploring the most critical parts of the very much larger space of what we are not good enough at. But is human intelligence truly general? The claim rests principally on our ability to learn, and this is certainly a core requirement for future intelligent systems. But we should also acknowledge that the human brain is the product of our particular evolutionary history and sports the evidence of its contingencies in many kluges, biases and peculiarities (Marcus, 2009). It would be reasonable to suppose that other more efficient, more general, more powerful and less flawed designs are possible.

Obviously there is still an immense amount to be learned about how human intelligence actually works and how the detailed structure and architecture of the brain produces it. And there will certainly be many insights that can be implemented in novel AI developments—for example the recent breakthroughs in understanding the workings of the neocortex (Hawkins et al., 2019), and the comprehensive program to develop cognitive models of how humans build compositionally structured causal models of the world grounded in their capacities for intuitive physics and intuitive psychology, so as to apply them to development of advanced AI systems (Tenenbaum et al., 2011; Lake et al., 2017). However, there is also an argument to be made that relying too much on guiding further development of AI on what is known about human intelligence, risks reproducing some of its limitations, or at least misses opportunities to deliberately and specifically mitigate them so as to extend and complement human capability.

## Desiderata for an AI Decision Support System for Complex Problems

The preceding discussion suggests an AI decision support system with three functional areas: an interface through which the human decision-maker interacts with it, the AI core generating and operating on a virtual conceptual model, and an interface to the outside world through which the AI core can grow its capability. Since the future system envisaged here is well beyond what is currently possible, its design can only be sketched out conceptually. The following two subsections offers some high level desiderata for the interfaces and the core, based on a hypothetical use case: in the light of the research insights presented in the preceding section, what would be most useful to a well-intentioned human decision-maker faced with very complex situations to manage?

A third subsection raises some of the ethical issues that must be addressed if such a system is able to be built.

### Interfaces to the Human Decision-Maker and the Outside World

The decision-maker^5^ needs to be able to give the system some initial direction about the problem, its scope, context, and goals and then develop them through dialogue, with intuitive visualizations presented by the interface to anchor and stimulate his participation. As these take shape the dialogue should extend to exploration of possible actions and their consequences, the development of courses of action, the building of necessary support from stakeholders and eventually monitoring the implementation of decisions made, and revising all above as more is learned and as the situation evolves.

The way that these are presented should support human understanding of the emerging conceptual model of the problem and its context, implying an appropriate level of coarse-graining in terms of intuitively comprehensible parameters. In particular, the interface should expose both explicit and implicit assumptions in the conceptual model, and possible levers of action and their consequences, both in the intended pathways and in other pathways that may be stimulated, together with estimates of the degree of uncertainty and the risks resulting from the consequent ranges of possible outcomes.

To reduce risks and further develop the conceptual model, the ongoing dialogue between the human and the interface should be able to launch searches for more data, initiate probing actions, and pose and explore “what if?” and “how could?” questions.

Conflicts and trade-offs also need to be identified – both those that must be explicitly managed, such as the balance between long-term and short-term outcomes, competing interests between different agents, and conflicts between espoused values and/or principles, and those that are actually false dichotomies which should be resolved by supporting exploration of integrative solutions in their place.

Most importantly, to enable the necessary adaptive approach, the interface must not only continuously evolve the conceptual model, but also in parallel prompt and support a process of continuous co-evolution of the goals, data collection plan, and both the structure and implementation of the strategy.

The decision-maker needs to have confidence that the system is in fact presenting accurate and comprehensive information and making judgments in accord with a transparent and agreed set of goals, values and principles. This implies additional requirements with respect to visibility of the goals, values and principles on which it is operating, flagging of uncertainties and assumptions, and where possible testing them, and demonstration that it is using its searching and learning resources to improve its conceptual model so as to reduce risks and uncertainties, in other words, actively subjecting its critical aspects to severe testing, and generating an audit trail for decisions made in relation to every complex issue.

These considerations imply that both the interface and the conceptual model behind it must be open systems that permit evolution of the vocabulary of the interface and the semantic map to the ontology of the model.

Since the interface is also the locus of the metacognitive support that the system can provide to the decision-maker, its design must be informed by an understanding of human limitations and shortcomings.

In particular, and building on, but going beyond the currently established principles of human computer interaction, for which a vast literature exists^6^, the interface design should scaffold a human decision-maker who seeks to overcome the specific difficulties and obstacles discussed in the previous section. For example, the interface could monitor for the influence of unconscious biases in the decision-maker's actions, such as confirmation bias, framing and recency biases, loss/gain asymmetry and so on, flagging them for conscious attention and offering options for reducing them. It could also reduce anxiety stemming from ambiguity, by demonstrating that an effective risk management strategy is in place (i.e., that indicators of emerging risks are being monitored and averting or mitigating action plans are ready to be triggered), and anxiety stemming from information overload, by effective partitioning of the information assimilation workload. Shouldering the workload of maintaining as many alternative working hypotheses as necessary, and exposing and testing the implicit assumptions in each of them, would assist in reducing the danger of premature convergence to a narrow singular view of the problem and hence selecting an inadequate strategy.

Overall what is being described here is a cooperative system where learning and adaptation occurs at the levels of both the human and the AI support system. Importantly, it also occurs at the level of the combined system—the interface supporting the decision-maker's learning by setting the example of its own learning behavior, in particular by continuously making predictions based on its current conceptual model, monitoring for the real world outcomes and revising its models in the light of what has been learned, and the human decision-maker being willing to expose their reasoning and ideas and subject them to analysis in their dialogue with the AI support system.

Of course these hypothetical examples are illustrative rather than prescriptive, and certainly not comprehensive, but they can serve as an adequate starting place for an iterative and continuously learning design process for the interface. Future research will no doubt surface many further opportunities to enhance both human decision-making performance, and the decision performance of the combined human plus AI support system.

Irrespective of how this design research agenda might evolve in detail in the future, one important consequence that seems inevitable is that the system's interface to the outside world must be able to autonomously engage in all relevant aspects as a trusted partner or agent of the decision-maker. Therefore, to enable the scale, tempo and depth of testing and learning that is called for in dealing with multi-faceted and open-ended complex problems, the interface to the outside world must be essentially unfettered and support multiple simultaneous high bandwidth interactions, as well as robust and secure. This point will have repercussions in discussing ethical concerns.

### AI Core

These requirements imply that the AI core needs the ability to develop situational models of the complex problems to be managed, and as much of their context as necessary, and to evolve them in a real time loop through predictive processing (Clark, 2015) and updating, i.e., by monitoring relevant developments, using the current version of the model to predict expected consequences, comparing predictions to actual outcomes, and hence updating the models as a result of what is learned. This means that the models must be open systems so that their structures and composition can change as more is learned, and as the situation itself changes over time.

The models need to exist at multiple scales—from coarse resolution to as fine a level as is required to model the relevant entities and events (whether by bottom-up models or by machine learning from data), and include all the dimensions relevant to the necessary scales of representation and all significant outcome variables, all accessible levers of influence that could be exercised, all the causal and influence pathways that may lead to significant consequences, the causal and influence relationships between entities and events, within and across scales, and their time dependence.

Including all the significant outcome variables implies a detailed representation of how success and failure of the complex problem will be judged, as well as intermediate outcomes and indicators that signal which consequence pathways are activated.

Situation models with such wide scope will necessarily be hybrid models, containing many detailed components, plus representations of the interactions and interdependencies between the components. To support zooming between scales, the core will need the ability to extract human comprehensible coarse-grained models^7^ from the more detailed models, whether data driven ML models or bottom-up micro-parameter based models.

To deal with complex problems at the “wicked” end of the complexity scale the core will need to be able to model humans who are stakeholders or actors in the situation, so that their responses to interventions or external events can be anticipated, and combinations of incentives and compensatory measures can be discovered that have a chance of fostering enough consensus for effective action to be taken. These models will also have to be learned by predictive processing, and continuously tested and updated.

In particular, the core will need to develop very good models of the human decision-makers which it is supporting, so that it can learn to interact with them in a way that they will value and trust.

In summary, besides the requisite models, the AI core needs a number of intelligent functions to enable all the operations implied by the considerations above, and the ability to evolve these as well in the light of its experience and interactions with the decision-maker in order to improve its capability.

### Ethical Issues

If such an intelligent support system is ever built it will be extremely useful and powerful. How could misuse be prevented? This is a serious question which must be addressed at the earliest stages of development. Internationally agreed guidelines^8^ and regulation, and a set of safety standards to be met, together with public transparency of the setup and use of any such system would at least make it possible to monitor the known systems. Detecting covert systems is more challenging and may need to be part of an overall cybersecurity capability, along with ensuring security from malicious manipulation.

The requirement for the system to be an autonomous agent with broad unlimited access to the world for learning and testing purposes, will raise particular ethical issues not only with respect to privacy, but also with respect to the commonly expressed fear that as AI becomes more intelligent and powerful, it will become harder, if not impossible, to continue to exert human control over it. The need to allow it to become more autonomous and intelligent and situated in the real world in order to be sufficiently effective is at odds with one proposed safeguard measure—that of strictly limiting its access to other real world systems. Therefore, it will be essential to develop other approaches to ensure that it continues to serve human needs and interests. However, defining those needs and interests will be a challenging and controversial wicked problem in itself. All these considerations point to the importance and urgency of addressing the ethical issues at the earliest stages.

Transparency is a powerful aid to addressing some of the ethical issues with AI supported decisions that may have adverse impacts on individuals or groups. For example prejudicial bias introduced into machine learning systems through training data could be exposed through triangulation with independent data sources. Similarly, exposing the assumptions that are made in the conceptual model, together with the efforts that have been made to test them, and whatever evidences are available to support or refute them, would help ethics watchdogs do their job.

## Discussion

It was noted in the Introduction section that the mutual ratcheting of complexity and intelligence did not necessarily terminate in a plateau of dynamic equilibrium. Under the right conditions it could continue and accelerate.

The right conditions are that selection pressure for intelligence remains strong and that the evolutionary process is able to generate further improvements in intelligence.

This describes where we are today. We desperately need more powerful intelligence to navigate the perilous waters we find ourselves in, and we have spawned completely new channels of creating and evolving intelligence beyond those afforded purely by our own biology. And both processes are arguably accelerating. Therefore, we do not have the option of turning back.

But it does raise another serious ethical question: where will the ratcheting dynamic of complexity and intelligence lead us? Will AI-aided resolution (or at least diminution) of tomorrow's most serious global problems generate even more disastrously wicked problems in a chain of escalation that rapidly drives humans to irrelevance?

While we cannot rule out worst case fears, the preceding discussions suggest two considerations that give grounds for cautious optimism.

Firstly the “wickedness” of wicked problems is in large part due to the shrinking of the viable option space as more agents with diverse priorities acquire a veto stake in the decision process and so need to be simultaneously satisfied. But a future AI support system could ameliorate this problem, through its capabilities to model the different agents and to devise strategies to win them over—as has already been demonstrated several times recently in the manipulation of voter opinions and preferences (Burkell and Regan, 2019). Of course this also raises ethical concerns and there would need to be a code of conduct agreed that provided transparency and guidelines as to what was acceptable.

Secondly, the ratcheting of complexity observed so far has largely been driven by short-sighted “fixes” of perceived problems, without much consideration of longer term and wider scope consequences—hence inadvertently creating further problems. This is intrinsically the case in natural evolutionary processes, and also very much the case with human decision-makers due to their limited cognitive bandwidth. (A good example is the rapid evolution of mines that are more lethal and harder to detect being driven by researching and fielding better vehicle protection and mine detection systems.) Again, a future AI support system could potentially reduce the pace of ratcheting, by anticipating longer term and wider scope consequences, factoring them in to the evaluation of strategy options, and where necessary actively reducing unwanted consequences with further supplementary actions.

Of course there is also the possibility that the tide does not turn, but rather continues to pose growing threat levels. But then what choice do we have? The immediate global and national problems facing us are urgent and we need all the help we can get. If we decline the opportunity to develop such systems, we will in any case face escalating problems, which might now include opponents and vested interests armed with the very capabilities we declined.

This suggests that it is time to shift the balance of investment in AI research and development away from competing with humans and toward creating new cooperative partnerships with them, to extend and buttress our joint capability to manage the rafts of wicked problems that threaten us. It will involve developing many new aspects of AI capability, but every new capability we create will help generate the next. We are rushing into a future that we can barely imagine, but we need to look ahead with as much clarity as we can muster, embrace the present opportunity we have to shape the trajectory, and use it to face the risks.

Such a discourse should be taken into account in setting priorities for investing in AI research and in formulating guidelines, standards and regulatory frameworks, which must be continuously reviewed and updated as we learn more about what is possible, what is necessary and what is to be avoided.

## Author Contributions

A-MG contributed conception and design of the paper and wrote the manuscript.

## Conflict of Interest

The author declares that the research was conducted in the absence of any commercial or financial relationships that could be construed as a potential conflict of interest.

## Footnotes

^1^Briefly the ten hallmarks are: no definitive formulation; no stopping rule; solutions are not true-or-false, but better or worse; no immediate or ultimate test of a solution; every solution attempt is a “one-shot operation”; no well-described set of potential solutions or permissible operations; essentially unique; can be considered a symptom of another problem; many possible explanations; and the decision-maker has “no right to be wrong” because of the gravity of the consequences.

^2^Managing complex situations involves many decisions over an extended period, with the consequences of earlier ones impacting on the necessity or possibility of later ones, and affecting the trajectory of the situation. To come to grips with how decision-making behaviors shape outcomes it is important to conduct experiments for a long enough period to allow these consequences to develop and confront the decision-maker.

^3^In order to learn what decision-making behaviors are more effective, the degree of complexity of the experimental environment has to be tuned to the edge of human competence so that data can also be gathered about what does work.

^4^The behaviors were grouped in five categories: goal decomposition; collecting and organizing information; projection and planning; decision and execution; and meta-cognition.

^5^For simplicity here we assume a single decision-maker, recognizing that in a real world problem situation there will be many involved and that will necessitate further support requirements.

^6^See for example the scope of CHI '19- Proceedings of the 2019 CHI Conference on Human Factors in Computing Systems http://st.sigchi.org/publications/toc/chi-2019.html

^7^This has proven difficult so far but recent work in this paper, Mattingly et al. (2018) and references therein may provide a breakthrough, not only in generating a human-comprehensible coarse-grained model, but importantly in identifying the few “stiff” parameter combinations that characterize its emergent macro level properties.

^8^See Jobin et al. (2019) for a recent overview.
